# Increased LIGHT leading to sFlt-1 elevation underlies the pathogenic link between hydatidiform mole and preeclampsia

**DOI:** 10.1038/s41598-019-46660-4

**Published:** 2019-07-12

**Authors:** Takayuki Iriyama, Guan Wang, Midori Yoshikawa, Nobuko Mimura, Haruka Matsui, Seisuke Sayama, Keiichi Kumasawa, Takeshi Nagamatsu, Kaori Koga, Tomomi Kotani, Kaoru Niimi, Eiko Yamamoto, Rodney E. Kellems, Yang Xia, Yutaka Osuga, Tomoyuki Fujii

**Affiliations:** 1 Department of Obstetrics and Gynecology, Faculty of Medicine, The University of Tokyo, Tokyo, Japan; 2 Department of Obstetrics and Gynecology, Tianjin Central Hospital of Obstetrics and Gynecology, Tianjin, China; 3 Department of Obstetrics and Gynecology, Nagoya University Graduate School of Medicine, Nagoya, Aichi Japan; 4 Department of Healthcare Administration, Nagoya University Graduate School of Medicine, Nagoya, Aichi Japan; 5 Departments of Biochemistry and Molecular Biology, The University of Texas McGovern Medical School, Houston, TX USA

**Keywords:** Predictive markers, Endocrine reproductive disorders

## Abstract

Hydatidiform moles are known to pose an extremely high risk of severe early-onset preeclampsia if left untreated. TNF superfamily cytokine, LIGHT has recently been reported to contribute to pathophysiology of preeclampsia. The present study aimed to investigate the involvement of LIGHT in hydatidiform moles. We measured the serum levels of LIGHT and sFlt-1 by ELISA in 17 women with complete hydatidiform mole (HM) and 20 gestational-age-matched normal pregnant women (control). As a result, the serum LIGHT levels were significantly higher in HM as compared with those in control (69.9 ± 9.6 pg/ml vs 25.4 ± 5.3 pg/ml, p = 0.0001) and the serum levels of LIGHT were significantly positively correlated with those of sFlt-1 in HM (r = 0.68, p = 0.0029). Immunohistochemical analysis revealed that the expression levels of LIGHT were increased in HM placentas as compared with controls, and LIGHT and sFlt-1 were co-localized in the trophoblast cells of HM. *In vitro* studies using primary syncytiotrophoblast cells demonstrated that LIGHT directly induced sFlt-1 expression in trophoblast cells. Our results indicated that elevated LIGHT in the trophoblast cells of hydatidiform mole induces sFlt-1, which might underlie the pathogenic mechanism of early-onset preeclampsia developing secondary to molar pregnancies.

## Introduction

In both hydatidiform moles and preeclampsia (PE), various maternal symptoms arise from placental abnormalities. Hydatidiform moles are known to pose a high risk of early-onset PE if the pregnancy continues with the moles left untreated. PE has been reported to develop as early as the 2nd trimester in 30–40% of pregnancies with untreated hydatidiform moles^1,2^. However, there have been very few studies on the molecular mechanisms that link hydatidiform moles with PE; much regarding these mechanisms remains unknown.

Many recent studies have demonstrated that placental dysfunction underlies the development of PE^3,4^. Due to hypoxia elicited by failure of uterine spiral artery remodeling, anti-angiogenic factors produced by trophoblast cells enter the maternal blood and induce PE symptoms. The most intensively researched anti-angiogenic factor has been soluble fms-like tyrosine kinase-1 (sFlt-1). In PE patients, blood levels of sFlt-1 increase prior to the onset of PE; therefore, sFlt-1 is considered a promising biomarker of PE^5^. Intriguingly, enhanced expression of sFlt-1 has also been reported in the blood and placenta of patients with hydatidiform moles^6,7^. These findings suggested that sFlt-1 may be involved in the underlying pathophysiological mechanism of PE subsequent to hydatidiform mole. The above findings also supported the hypothesis that the placental dysfunction plays a central role in the development of early-onset PE. Therefore, investigation of the pathological mechanisms associated with the development of maternal PE symptoms in hydatidiform mole may lead to the further clarification of the pathophysiology of placental abnormalities related to PE.

Enhanced maternal inflammation, which is characterized by increased expression of inflammatory cytokines, is strongly associated with the onset of PE^3,8^. LIGHT, tumor necrosis factor (TNF) superfamily member 14, has recently been reported to be a key inflammatory cytokine in the pathogenesis of PE, due to its enhanced expression in the blood and placenta of PE patients^9^. LIGHT not only elicits the local effects of placental damage, but is also involved in the manifestation of systemic PE symptoms by inducing the placental increased expression of sFlt-1^10^. The mechanism by which anti-angiogenic factors and inflammatory cytokines produced in the placenta lead to PE has been largely clarified, however, little is known regarding the mechanism by which the placental damage is triggered. One reason for this lack of knowledge is that it is impossible to investigate the placenta prior to the onset of PE symptoms. Analysis of placental tissue in hydatidiform mole patients who are considered to be in a presymptomatic state of early-onset PE, which is caused by placental damage, could play a major role in identifying the mechanism of onset of placental damage in PE. Therefore, the present study aimed to provide novel evidence regarding the pathogenic mechanisms of PE by examining the association between sFlt-1 expression and LIGHT in patients with hydatidiform moles.

## Results

### Serum levels of LIGHT are increased and positively correlated with those of sFlt-1 in patients with hydatidiform mole

**Figure 1:**
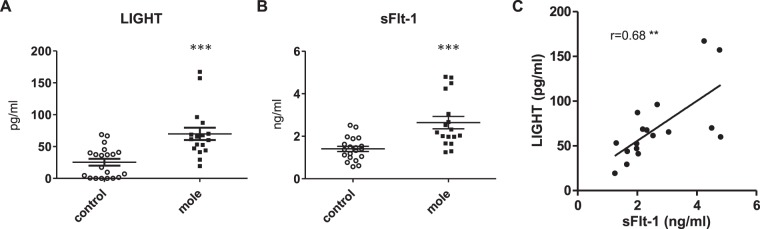
Circulating levels of LIGHT and sFlt-1 in hydatidiform mole patients and gestational age-matched normal pregnant women. (**A**) Serum levels of LIGHT in complete hydatidiform mole group (mole: n = 17) and normal pregnancy group (control: n = 20) were determined by ELISA. ***P = 0.0001 vs control. (**B**) Serum levels of sFlt-1 in mole (n = 17) and control (n = 20) were determined by ELISA. ***P = 0.0001 vs control. (**C**) Serum levels of LIGHT and sFlt-1 in mole (n = 17) were positively correlated. Spearman’s rank correlation coefficient (r) was 0.68. **P = 0.0029.

As shown in Table 1, background clinical characteristics including the gestational age were comparable between hydatidiform mole patients and non-molar pregnant women controls included in this study. To examine the levels of LIGHT and sFlt-1 in the serum of patients with hydatidiform mole and gestational-age-matched control group, we utilized a sensitive ELISA. We found that the serum LIGHT levels were significantly higher in patients with hydatidiform mole compared with those in non-molar pregnant women (69.9 ± 9.6 pg/ml vs 25.4 ± 5.3 pg/ml, *P* = 0.0001) (Fig. 1A). As shown in Fig. 1B, serum sFlt-1 levels in patients with hydatidiform mole were also higher than those in non-molar pregnant women (2.64 ± 0.29 ng/ml vs 1.41 ± 0.12 ng/ml *P* = 0.0002). Furthermore, the serum levels of sFlt-1 were positively correlated with those of LIGHT in mole group as shown in Fig. 1C (r = 0.68, p = 0.0029).

**Table 1:** Clinical characteristics for human subjects.

	Control (n = 20)	Mole (n = 17)
Age (yr), mean ± SEM	32.5 ± 0.8	31.7 ± 1.7
Gestational age (week), mean ± SEM	9.6 ± 0.1	9.0 ± 0.38
Maternal complication		
Obesity (BMI > 25 kg/m^2^)	0	0
Hypertension	0	0
Diabetes mellitus	0	0

### Increased expression of LIGHT in the villous tissues of patients with hydatidiform mole

**Figure 2:**
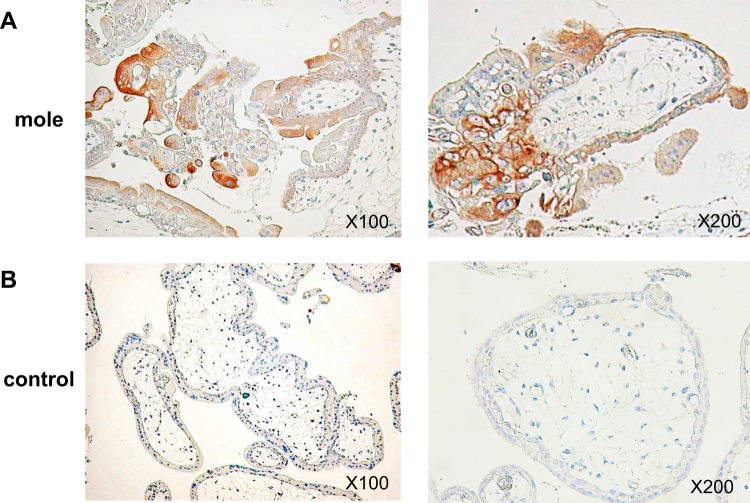
Increased expression of LIGHT in molar placentas. Representative images of immunohistochemical staining for LIGHT in the placenta of a hydatidiform mole case (**A**) and normal control (**B**) are shown.

We next examined the expression profiles of LIGHT in the villous tissues of hydatidiform mole patients and gestational-age-matched non-molar pregnant women controls using immunohistochemistry. Immunohistochemical analysis demonstrated that the protein expression levels of LIGHT in the villous tissues were increased in hydatidiform mole patients compared with normal control (Fig. 2A,B). LIGHT protein expression was clearly present especially in syncytiotrophoblast cells in the hydatidiform mole samples. LIGHT expression was also detected in cytotrophoblast cells and decidual cells of hydatidiform mole. By contrast, LIGHT staining in control samples was barely detectable in syncytiotrophoblast cells and the underlying cytotrophoblast cells under the same staining conditions (Fig. 2B). These results indicated that the increased expression of LIGHT in trophoblast cells, especially in the syncytiotrophoblast cells facing maternal blood, might contribute to the elevation of serum LIGHT levels in hydatidiform mole patients.

### Co-localized expression of LIGHT and Flt-1/sFlt-1 in the villous tissues of hydatidiform mole

**Figure 3:**
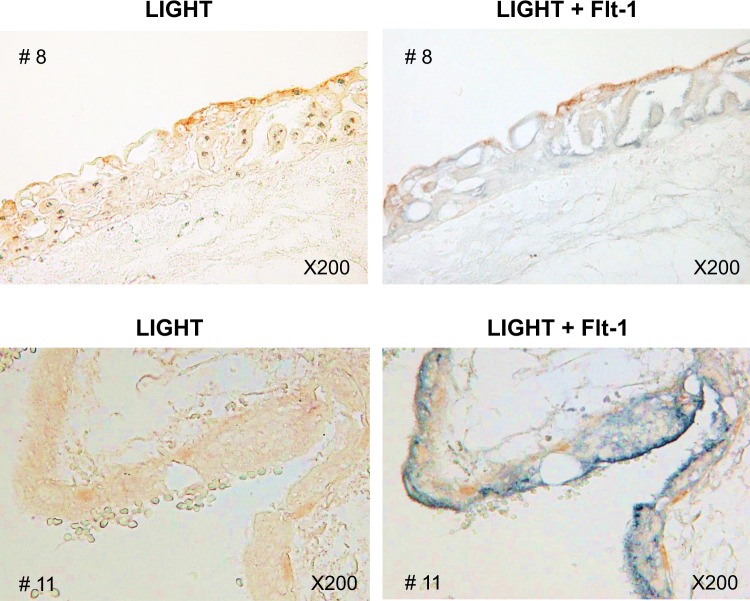
Co-localized expression of LIGHT and Flt-1/sFlt-1 in molar placentas. Expression of LIGHT and Flt-1/sFlt-1 were sequentially detected using anti-LIGHT antibody visualized with diaminobenzidine (brown), followed by anti-Flt-1/sFlt-1 antibody visualized with SG peroxidase substrate (blue). Representative images of two cases (#8 and #11) are shown.

Next, to link the correlative expression of LIGHT and sFlt-1 in the blood to that in the villous tissue of hydatidiform mole, we conducted immunohistological double staining of LIGHT and sFlt-1. As previously reported^7^, Flt-1/sFlt-1 expression is clearly apparent in trophoblast cells of hydatidiform mole (Fig. 3). Double staining of LIGHT and Flt-1/sFlt-1 revealed that LIGHT and sFlt-1 are co-localized in trophoblast cells of hydatidiform mole.

### LIGHT directly induces sFlt-1 expression in trophoblast cells

To investigate the mechanism underlying positively-correlated serum levels and co-localization of LIGHT and sFlt-1, we examined whether LIGHT is capable of inducing sFlt-1 in trophoblast cells.

**Figure 4:**
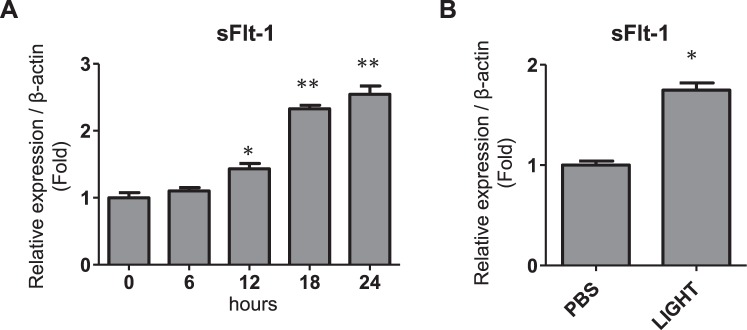
LIGHT induces sFlt-1 expression in human trophoblast cells. (**A**) HTR-8/SVneo cells were treated with 100 pg/ml recombinant human LIGHT under ambient oxygen levels. mRNA was extracted from cells, and sFlt-1 mRNA levels were determined by real time RT-PCR (n = 4 each). *P < 0.05, **P < 0.01 vs base line (0 hour). (**B**) Cultured syncytiotrophoblast cells were treated with 100 pg/ml LIGHT for 18 hours under ambient oxygen levels. mRNA was extracted from cells, and sFlt-1 mRNA levels were determined by real time RT-PCR (n = 3 each). *P < 0.05 vs PBS.

First, human trophoblast cell line HTR-8/SVneo cells were treated with LIGHT under ambient oxygen levels, and sFlt-1 mRNA expression was determined by real-time RT-PCR. As shown in Fig. 4A, LIGHT treatment induced increased sFlt-1 mRNA production in HTR-8/SVneo cells in a time-dependent manner. To examine the impact of LIGHT on syncytiotrophoblast cells in which both of LIGHT and sFlt-1 were highly expressed, we utilized primary syncytiotrophoblast cell culture obtained by spontaneous differentiation from cytotrophoblast cells isolated from human term placentas as previously reported^11^. As a result, we found that sFlt-1 mRNA was significantly induced by the treatment of LIGHT in syncytiotrophoblast cells (Fig. 4B). These results provide a valuable evidence that LIGHT is closely associated with the induction of sFlt-1 expression in trophoblast cells, and LIGHT might play a key role in the overproduction of sFlt-1 in hydatidiform mole patients.

## Discussion

The molecular mechanism of PE onset associated with hydatidiform moles is largely unknown. The only finding previously reported was that the expression of sFlt-1 is enhanced in the blood and placenta of patients with hydatidiform moles^6^. The present study demonstrated that the expression of LIGHT is elevated in the blood and placenta of hydatidiform mole patients. In blood, the expression of LIGHT was positively correlated with increases in levels of sFlt-1, while in the placenta, LIGHT was co-expressed with sFlt-1 in the trophoblast cells. These results suggested that the enhanced expression of sFlt-1 is closely associated with increased expression of LIGHT in hydatidiform mole patients. In addition, it was demonstrated in an *in vitro* experiment that LIGHT can directly induce the production of sFlt-1 in trophoblast cells, particularly in the syncytiotrophoblast cells.

Findings from previous studies led to the following prevailing model of the mechanism of PE pathology: placental dysfunction subsequent to impairment in placentation early in pregnancy leads to the release of molecules such as sFlt-1 from the placenta into maternal blood, thereby inducing maternal symptoms^3,4^. Little is known regarding impairment in placentation, despite its significant involvement in abnormalities of fetomaternal immunologic tolerance. A major factor in the lack of progress in identifying the mechanism that leads to impairment in placentation is that placental tissue cannot be examined prior to the onset of PE. It is incredibly difficult to identify the actual factors that elicit impairment in placentation beginning early in pregnancy. Hydatidiform moles have long been known to lead to early-onset PE in 30–40% of cases^1,2^. Placental damage is considered to play a particularly large role in the mechanism of early-onset PE. Therefore, analysis of the placenta in hydatidiform mole patients has the potential to be useful in identifying candidate factors involved in the onset of impairment of placentation in PE, as well as in searching for biomarkers of PE. In order to advance this potential, sFlt-1, which is involved in the pathological mechanism of PE starting from the onset of impairment of placentation, is now being clinically used as a marker for predicting the onset of PE; enhanced sFlt-1 expression has been reported in the blood and placenta of hydatidiform mole patients^6,7^.

Although the association between LIGHT signaling and obstetric disorders is largely unknown, enhanced signaling had recently been reported to be closely involved in the pathogenesis of PE^9^. In this report, elevated expression of LIGHT in the placentas of PE patients was particularly pronounced in the syncytiotrophoblast cells. This result is consistent with the present study, in which the expression of LIGHT was elevated in the syncytiotrophoblast cells of hydatidiform mole tissue. The syncytiotrophoblast cells are at the interface with maternal blood; the entry of its debris and various factors, such as sFlt-1 produced in the syncytiotrophoblast cells, into maternal blood is known to be deeply involved in the development of PE features^8^. Thus, the elevated expression of LIGHT in the syncytiotrophoblast cells of hydatidiform mole tissue supports the finding of elevated levels of LIGHT in blood. sFlt-1 also demonstrated markedly elevated expression in the trophoblast cells of hydatidiform mole tissue and coexisted with LIGHT. This finding suggests that LIGHT may be closely involved in sFlt-1 production and supports the finding that elevated levels of sFlt-1 and LIGHT in blood were positively correlated with each other. These results suggest that LIGHT could have a major role in the mechanism of PE, which frequently develops in patients with hydatidiform moles.

LIGHT is produced by cells of the innate and adaptive immune system, including granulocytes, monocytes, macrophages, dendritic cells, and T cells^12^. It is initially present on the cell membrane as a homotrimer, but can be released from the cell membrane via proteolytic cleavage and exist in a soluble form. Whether membrane bound or free, LIGHT can bind to two receptors, herpes virus entry mediator (HVEM), which is present on T cells, and lymphotoxin β receptor, present on non-lymphoid hematopoietic cells including natural killer cells. Downstream signaling occurs through activation of the NF-κB pathway, a common feature of TNF family cytokines. LIGHT‐mediated activation of HVEM receptors on T cells results in a costimulatory response leading to T‐cell activation, cell proliferation, and cytokine production. HVEM and lymphotoxin β receptors are also present at elevated levels on trophoblast cells, where LIGHT stimulates increased production of hypoxia inducible factor 1-α (HIF-1α), a transcription factor responsible for the regulation of a number of genes^9,10,13^. A causal role for LIGHT in features of PE is supported by experiments showing that LIGHT is able to induce hypertension when introduced into pregnant or non-pregnant mice^9^. Recent evidence indicates LIGHT-induced hypertension requires tissue transglutaminase, a widely distributed enzyme that catalyzes the posttranslational modification glutamine residues on proteins^14^. In summary, LIGHT has emerged as a key factor involved in a number of disease conditions including not only PE, but also cancer, hepatitis, asthma, and autoimmune diseases^15–18^. Further studies are required to understand the mechanisms responsible for increased LIGHT production in women with hydatidiform mole and PE.

In PE, production of sFlt-1 is believed to occur primarily in the placenta^19^. The primary cause of sFlt-1 overproduction in the placenta is considered to be placental hypoxia associated with placentation failure; however, many studies have reported that sFlt-1 is also produced by trophoblast cells under various stress conditions independent of hypoxia, including inflammatory cytokines^10,20^. In a previous report using placental villous explants^10^, LIGHT was shown to induce HIF-1α independent of hypoxia and promote production of sFlt-1. A placental villous explant culture system contains not only trophoblast cells, but a mixture of various types of cells, including stromal cells and endothelial cells. Therefore, in order to conduct an assessment in pure trophoblast cells, we used a human trophoblast cell line, HTR-8/SVneo cells, and a primary culture of syncytiotrophoblast cells, which we recently established and reported^11^. Consequently, we show here that LIGHT can induce sFlt-1 independent of hypoxia, not only in HTR-8/SVneo cells, but also in syncytiotrophoblast cells. This result supports the association between LIGHT and elevated sFlt-1 expression observed in hydatidiform mole tissue. The above result also demonstrates that LIGHT is directly involved in the induction of sFlt-1 production in trophoblast cells and is closely associated with the pathogenesis of PE. LIGHT and its receptors are reportedly expressed in syncytiotrophoblast cells of the first trimester placentas^13,21^. Mechanistically, we speculate that upregulated HIF-1α through enhanced LIGHT signaling via its receptors is involved in sFlt-1 overproduction and in the pathogenesis of PE features seen in hydatidiform mole patients. Taken together, the interrelationship between LIGHT and sFlt-1 is strongly suggested to be a crucial factor in the pathogenesis of PE triggered by placental damage.

In a prior study, elevated blood levels of TNF-α and other cytokines in hydatidiform moles were associated with their development into trophoblastic tumors^22^. The association of LIGHT levels and sFlt-1 expression with subsequent development into trophoblastic tumors could not be sufficiently examined because only 2 cases developed into invasive moles in the current study. LIGHT has been reported to activate anti-tumor immunity and inhibit the progress of cancer^18,23^. In addition, sFlt-1 has been reported to exert antitumor effects (namely, cell proliferation inhibition, cell death, and angiogenesis inhibition) in various types of cancer^24^. Elevated levels of sFlt-1 associated with elevated levels of LIGHT might in fact be involved in the development of trophoblastic disease symptoms in an inhibitory manner. Examination of larger numbers of cases may reveal the precise association.

The present study demonstrated that the expression of LIGHT is enhanced in the blood and placenta of hydatidiform mole patients, and that this enhanced expression is closely associated with elevated sFlt-1 levels. In addition to indicating the importance of LIGHT in the pathogenic mechanisms of PE subsequent to hydatidiform mole development, these results also suggest that LIGHT is involved in placental damage leading to placental dysfunction, which can lead to the onset of PE, and is closely associated with the development of PE. The above results also suggest that LIGHT, similar to sFlt-1, might have the potential to serve as a biomarker for predicting the onset of PE.

## Methods

### Patients

This study was conducted under the approval of the institutional review board of our institution (11542) and Nagoya University Hospital (2017-0208), and all experiments were conducted in accordance with relevant guidelines. Hydatidiform mole patients and normal control pregnant women participating in other studies approved by the IRB of our institution (2914) and Nagoya University (2014-009-3) with the signed informed consent were enrolled in this study. We made public announcements for those participants on the internet homepage of our institution and Nagoya University Hospital, in accordance with the request of ethics committee and guidelines. Seventeen women with complete hydatidiform mole and 20 gestational-age-matched pregnant women who did not display any complication in subsequent pregnancy courses were included in this study for the analysis of blood samples. Background clinical characteristics of hydatidiform mole patients and normal pregnant women included in this study are shown in Table 1. Blood samples were obtained between 6 to 13 weeks of gestation before the evacuation of hydatidiform mole. All mole cases were pathologically diagnosed as complete hydatidiform mole and absence of a coexistent fetus by expert pathologists. Normal placentas were obtained from women who underwent induced abortion during the first trimester.

### Measurement of serum levels of LIGHT and sFlt-1 by ELISA

The concentration of LIGHT and sFlt-1 in serum was quantitatively measured by ELISA kits which are commercially available (R&D) as previously described^25^.

### Immunohistochemical staining for LIGHT

Tissue sections were de-paraffined with xylene, and rehydrated through an ethanol series and TBS. Antigen retrieval was performed by microwave treatment, with Citrate buffer, pH6. Endogenous peroxidase was blocked with 0.3% H_2_O_2_ in methanol for 30 min, followed by incubation with Protein Block (Genostaff) and avidin/biotin blocking kit (Vector). The sections were incubated with anti-LIGHT(TNFSF14) rabbit polyclonal antibody (SIGMA) at 4 °C overnight. They were incubated with biotin-conjugated goat anti-rabbit Ig (Dako), for 30 min at RT, followed by the addition of peroxidase conjugated streptavidin (Nichirei) for 5 min. Peroxidase activity was visualized by diaminobenzidine. The sections were counterstained with Mayer’s Hematoxylin (MUTO), dehydrated, and then mounted with Malinol (MUTO).

### Immunohistochemical double staining for LIGHT and Flt-1

For the first staining, antigen retrieval was performed by microwave treatment, with citrate buffer, pH6.0. Endogenous peroxidase was blocked with 0.3% H_2_O_2_ in methanol for 30 min, followed by incubation with Protein Block (Genostaff). The sections were incubated with anti-LIGHT (TNFSF14) rabbit polyclonal antibody (SIGMA) at 4 °C overnight. They were incubated with Histofine simple stain MAX-PO(R) (Nichirei), for 30 min at RT. Peroxidase activity was visualized by diaminobenzidine. For the second staining, endogenous peroxidase was blocked with 0.3% H_2_O_2_ in methanol for 30 min, followed by incubation with Protein Block (Genostaff) and avidin/biotin blocking kit (Vector). The sections were incubated with anti-VEGFR1 goat polyclonal antibody (R&D) at 4 °C overnight. They were incubated with biotin-conjugated horse anti-Goat IgG (Vector), for 30 min at RT, followed by the addition of peroxidase conjugated streptavidin (Nichirei) for 5 min. Peroxidase activity was visualized by SG peroxidase substrate (Vector). The sections were mounted with G-Mouse (Genostaff) and Malinol (MUTO).

### Cell culture

HTR-8/SVneo cells were cultured on 24-well plates with Dulbecco’s modified Eagle medium (DMEM, Wako) with 10% fetal bovine serum (FBS) and antibiotic-antimycotic solution at 37 °C under 5% CO_2._ Human villous cytotrophoblast cells were isolated from normal term placentas, and *in-vitro* syncytialization of isolated cytotrophoblasts were conducted as previously reported^11^. Briefly, only HLA-ABC negative cells were collected using a Mini MACS TM separator (Miltenyi Biotec). Purified cytotrophoblasts were diluted in Iscove’s Modified Dulbecco’s Medium (IMDM) (GE Healthcare) supplemented with 10% fetal bovine serum (FBS), 1% Antibiotic-Antimycotic (Thermo Fisher Scientific), 200 mM L-glutamine and 10 ng/ml epidermal growth factor (EGF). After seeding the isolated cytotrophoblasts, spontaneous cell fusion occurred simultaneous with syncytialization *in vivo*. After 96 hours in culture, the culture wells were covered by a monolayer of syncytialized trophoblast, then treated with 100 pg/ml recombinant human TNFSF14/LIGHT (R&D) for 18 hours.

### Real time RT-PCR analysis

RNA was isolated and real time RT-PCR was conducted as previously described^10^. Syber green was utilized for analyzing genes by using following primers: Human sFlt-1-F (5′-ACAATCAGAGGTGAGCACTGCAA-3′), sFlt-1-R (5′-TCCGAGCCTG AAAGTTAGCAA-3′), Human β-actin-F (5′-CATGTACGTTGCTATCCAGGC-3′), β-actin-R (5′-CTCCTTAATGTCACG CACGAT-3′).

### Statistical analysis

Data values are expressed as mean ± SEM. All data were subjected to statistical analysis using Mann-Whitney’s *U* test in two-group analysis, or one-way analysis of variance followed by the Newman-Keuls post hoc test in multi-group analysis. Correlation analysis was conducted using Spearman’s rank-order correlation. Statistical significance was set at P < 0.05 and analyzed using GraphPad Prism 7 (GraphPad).
